# Investigating the Equivalent Plastic Strain in a Variable Ring Length and Strut Width Thin-Strut Bioresorbable Scaffold

**DOI:** 10.1007/s13239-022-00625-3

**Published:** 2022-07-11

**Authors:** Ben Hoddy, Naveed Ahmed, Kadem Al-Lamee, Nial Bullett, Nick Curzen, Neil W. Bressloff

**Affiliations:** 1 Computational Engineering and Design Research Group, University of Southampton, Southampton, UK; 2 Arterius Ltd, Leeds, UK; 3 Coronary Research Group, Southampton University Hospitals NHS Trust, Southampton, UK; 4 Faculty of Medicine, University of Southampton, Southampton, UK

**Keywords:** Scaffold, Bioresorbable, PLLA, FEA, PEEQ

## Abstract

**Purpose:**

The ArterioSorb$^{\rm{{TM}}}$ bioresorbable scaffold (BRS) developed by Arterius Ltd is about to enter first in man clinical trials. Previous generations of BRS have been vulnerable to brittle fracture, when expanded *via* balloon inflation *in-vivo*, which can be extremely detrimental to patient outcome. Therefore, this study explores the effect of variable ring length and strut width (as facilitated by the ArterioSorb$^{\rm{{TM}}}$ design) on fracture resistance *via* analysis of the distribution of equivalent plastic strain in the scaffold struts post expansion. Scaffold performance is also assessed with respect to side branch access, radial strength, final deployed diameter and percentage recoil.

**Methods:**

Finite element analysis was conducted of the crimping, expansion and radial crushing of five scaffold designs comprising different variations in ring length and strut width. The Abaqus/Explicit (DS SIMULIA) solution method was used for all simulations. Direct comparison between *in-silico* predictions and *in-vitro* measurements of the performance of the open cell variant of the ArterioSorb$^{\rm{{TM}}}$ were made. Paths across the width of the crown apex and around the scaffold rings were defined along which the plastic strain distribution was analysed.

**Results:**

The *in-silico* results demonstrated good predictions of final shape for the baseline scaffold design. Percentage recoil and radial strength were predicted to be, respectively, 2.8 and 1.7 times higher than the experimentally measured values, predominantly due to the limitations of the anisotropic elasto-plastic material property model used for the scaffold. Average maximum values of equivalent plastic strain were up to 2.4 times higher in the wide strut designs relative to the narrow strut scaffolds. As well as the concomitant risk of strut fracture, the wide strut designs also exhibited twisting and splaying behaviour at the crowns located on the scaffold end rings. Not only are these phenomena detrimental to the radial strength and risk of strut fracture but they also increase the likelihood of damage to the vessel wall. However, the baseline scaffold design was observed to tolerate significant over expansion without inducing excessive plastic strains, a result which is particularly encouraging, due to post-dilatation being commonplace in clinical practice.

**Conclusion:**

Therefore, the narrow strut designs investigated herein, are likely to offer optimal performance and potentially better patient outcomes. Further work should address the material modelling of next generation polymeric BRS to more accurately capture their mechanical behaviour. Observation of the *in-vitro* testing indicates that the ArterioSorb$^{\rm{{TM}}}$ BRS can tolerate greater levels of over expansion than anticipated.

## Introduction

Bioresorbable scaffolds (BRS) offer several potential benefits over conventional metallic stents including a reduced requirement for antiplatelet pharmacology, the reduction of late thrombosis, restoration of physiological vasomotion and reduced complication of repeat intervention.^[3,10]^ To date, first generation BRS have not demonstrated any significant advantages over drug eluting stents (DES), and, in fact, have so far reported higher incidence of device thrombosis until 3 years post intervention. However, between three and 5 years post intervention, the event rates were not significantly different from DES, and fewer scaffold thrombosis were reported.^[10,21,22]^ A possible explanation for this performance relates to design constraints. Specifically, given the mechanical inferiority of polymeric materials (lower elastic stiffness and yield strength) compared to metals, the use of thick struts, often in excess of 150 ${\upmu }$m was necessary in early generations of BRS. Fracture of the scaffold struts, post implantation, was also regularly reported in first generation devices due to the brittle behaviour of BRS, particularly compared with metallic DES.^[15,34]^ Therefore, the challenge for the next generation of BRS, some of which have already provided promising initial data,^[20,23,36]^ lies in reducing strut thickness without loss in mechanical performance, particularly radial strength, as well as facilitating large over expansions during post-dilatation without inducing strut fracture.^[6]^

Another important consideration for all coronary scaffolds is side-branch access.^[27]^ Given the recommended ‘provisional stenting’ technique for coronary bifurcations,^[4]^ it is important that the deformation of struts precluding side-branches is reduced to minimise the stress induced on the vessel wall. Excessive damage to the vessel walls caused by the placement of the stent/scaffold is commonly linked with poor clinical outcomes, particularly restenosis,^[16,19]^ due to the onset of neointimal hyperplasia.^[14,24]^ Maximising the open cell area of the scaffold could help to achieve a reduction in damage to the vessel wall.

The equivalent plastic strain, *PEEQ*, developed in the struts of BRS in balloon expansion is a scalar metric that provides insight into the state of plastic deformation in a structure. Plastic strain is inherently relied upon by coronary stents/scaffolds to maintain their target diameter in the diseased vessel and avoid excessive elastic recoil and so this metric will help determine the mechanical performance of BRS. Migliavacca *et al*.^[26]^ investigated the effect of a number of design variables on the performance of a slotted tube stent, including consideration of the *PEEQ*. However, only the total *PEEQ* was considered rather than its distribution across the scaffold struts. Additionally, Wang *et al*.^[37]^ optimised the design of a BRS by seeking to minimise the concentration of *PEEQ* at the crown apex to homogenise the strain distribution and related the *PEEQ* observed in *silico* to *in-vitro* observations of stress crazing, first reported by Radu *et al*.^[33]^ We propose that *PEEQ* is an important but relatively poorly understood metric in the case of coronary scaffolds as it is rarely considered in the literature. Assessment of the *PEEQ* will help to provide insight into the avoidance of brittle strut fracture. Excessive levels of strain, a surrogate of *PEEQ*, will lead to strut fracture whilst insufficient *PEEQ* will result in higher levels of elastic recoil, due to the limited amount of permanent deformation in the scaffold struts. Therefore, understanding the relationship between scaffold geometry, *PEEQ* and mechanical performance is extremely important. Specifically, understanding the distribution of *PEEQ* across the crown width and around the scaffold ring, where *PEEQ* is greatest is critical.

The ArterioSorb$^{\rm{{TM}}}$ BRS is an open cell thin-strut coronary scaffold manufactured by solid phase orientation of poly-l-lactic acid (PLLA) ^[2,11]^ It comprises a single closed cell at the central rings with subsequent open cells either side. Whilst the original BRS design incorporated variable ring lengths, this work investigates the combined effects of variable ring length and variable strut width. No other study appears to have reported these design variables to explore the mechanical performance of a BRS and improve the associated fracture resistance. Therefore, we utilised geometry control to investigate the distribution of equivalent plastic strain in the scaffold struts of five different designs, representing modifications to a baseline design (referred to as design 5) which is based upon the open-cell ArterioSorb$^{\rm{{TM}}}$ design. Finite element analysis (FEA) was used to simulate the crimping, balloon expansion and radial strength testing which mimicked the *in-vitro* testing of scaffolds by Arterius Ltd. Results from the bench testing of the baseline design were used to validate the *in-silico* data. Over expansion of the baseline design as well as the expansion of previous generations of the ArterioSorb$^{\rm{{TM}}}$ BRS were considered to provide further insight into BRS mechanical behaviour, particularly related to the adverse effects observed in the over expansion of BRS.

Specific radial strength (*SRS*), recoil ($R_{\%}$), cell area (*CA*) and final diameter (*FD*) were considered along with average maximum *PEEQ* in the crowns of the scaffold end rings ($PQ_{\rm{{max}}}$) to appraise the scaffold performance. Also, paths in particular locations on the scaffold geometry were defined along which the equivalent plastic strain was observed to aid understanding of its distribution across critical locations in the scaffold.

## Methodology

### 3D Geometry

**Figure 1:**
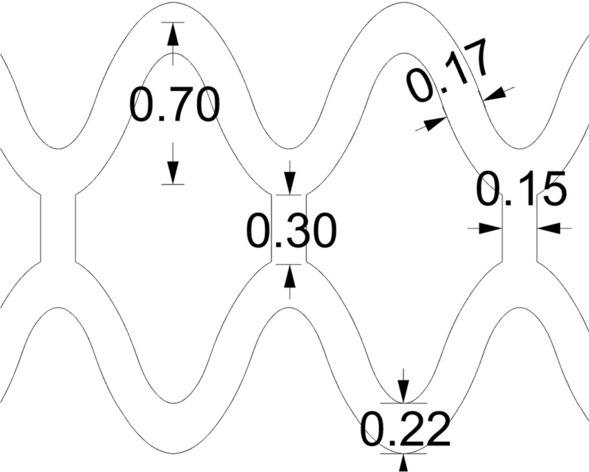
Central, closed ring geometry for a single repeating unit, based upon the ArterioSorb$^{\rm{{TM}}}$ BRS. The width at the crown apex is 0.22 mm, the strut width is 0.17 mm, the ring length is 0.7 mm, the connector length is 0.3 mm and the connector width is 0.15 mm.

The geometry of the central closed ring, configured to represent the ArterioSorb$^{\rm{{TM}}}$ BRS^[11]^ is shown in Fig. 1. The scaffold has a constant ring length of 0.7 mm and a strut width of 0.17 mm increased to 0.22 mm at the crown apex. The shape of the rings is defined by a cosine function, defined as:

1 $$\begin{aligned} y = L_{\rm{{Ring}}}cos(\omega x) \end{aligned},$$

where the parameter $L_{\rm{{Ring}}}$ denotes the ring length and $\omega$ is the phase parameter, adjusted such that an outer diameter (OD) of 2.54 mm is achieved using eight crown units. The values of *x* and *y* give the position in the circumferential and axial directions, respectively. This function defines the spine of the scaffold, over which the crown and strut widths are laid. The scaffold consists of closed cells between its two central rings with open cells located either side.

To explore variable ring length and strut width designs, design variables are defined for the ring length factor ($F_{\rm{{RL}}}$) and strut width factor ($F_{\rm{{SW}}}$), to define the rates of change of the ring length and strut width ($S_{\rm{{width}}}$). $L_{\rm{{Ring}}}$ and $S_{\rm{{width}}}$ for each ring are given by:

2 $$\begin{aligned} L_{\rm{{Ring}}}= & {} 0.7(F_{\rm{{RL}}})^{n} \end{aligned},$$

3 $$\begin{aligned} S_{\rm{{width}}}= & {} 0.17(F_{\rm{{SW}}})^{n} \end{aligned},$$

where *n* denotes the ring number which is zero at the central closed ring and increases symmetrically towards the rings at either end of the scaffold. The parameterisation of the geometry is such that the inner radius of the crown is not fixed. Therefore, as $F_{\rm{{SW}}}$ is increased, a wider crown results in a smaller radius of curvature at the inside of the crown.

**Figure 2:**
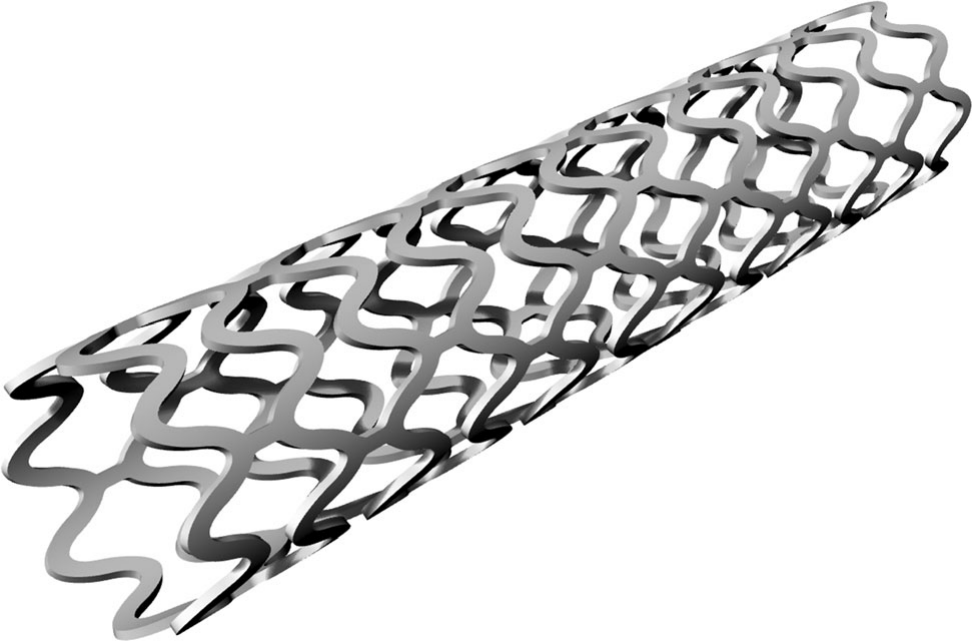
3D geometry based upon the ArterioSorb$^{\rm{{TM}}}$ BRS. The baseline scaffold design consists of 12 rings, has a length of 12.95 mm, an OD of 2.54 mm and thickness of 0.095 mm. The ring length and strut width remains constant along the scaffold length as both $F_{\rm{{RL}}}$ and $F_{\rm{{SW}}}$ are unity.

The following parameters remain constant across all designs; ring length of the central closed rings (0.7 mm, measured peak to trough for the underlying spine of the scaffold), ratio of the crown to strut width (220/170), thickness in the radial direction (0.095 mm), number of rings (12) and nominal diameter (2.54 mm). The 3D geometry of the baseline scaffold design is shown in Fig. 2.

Five scaffold designs were investigated, with the baseline design referred to as design 5. The design variables were chosen such that the ring length and strut width at the end rings were not excessively large or small for the scaffolds’ intended expansion diameter of 3.8 mm. Table 1 presents the geometrical parameters for each of the designs as well as the dimensions at the scaffold end ring.

**Table 1:** Geometrical parameters of the variable ring-length and strut-width BRS for the five designs investigated, including the baseline scaffold that was also tested *in-vitro* (design 5).

Design number	Ring length factor $F_{\rm{{RL}}}$	Strut width factor $F_{\rm{{SW}}}$	Ring length (end ring) (mm) $L_{\rm{{Ring}}}$	Strut width (end ring) (mm) $W_{\rm{{Strut}}}$
1	0.96	0.96	0.285	0.179
2	0.96	1.04	0.285	0.268
3	1.04	0.96	0.426	0.179
4	1.04	1.04	0.426	0.268
5 (baseline)	1.00	1.00	0.350	0.220

### Constitutive Material Model

The extruded PLLA tubes from which the polymer backbone of the ArterioSorb$^{\rm{{TM}}}$ is manufactured are pre-processed using biaxial die drawing whereby the tube is heated above its glass transition temperature on an expanding mandrel. This induces crystallinity and orientates the polymer chains in both the axial and circumferential directions. As a result, the PLLA displays significant anisotropy with higher strength and stiffness in the axial direction, due to increased polymer chain crystalinity in this direction. The anisotropic plastic potential material model, as used by Pauck and Reddy^[31]^ and Blair *et al*.,^[7]^ utilises stress-strain data obtained by Arterius Ltd (Leeds, UK) from uniaxial tensile tests of dogbone shaped specimens cut in both the axial and circumferential directions from PLLA tubes.

The material’s elastic behaviour is completely described by the bulk modulus (K) and the shear modulus ($\hat{G}$), defined as:

4 $$\begin{aligned} K= & {} \frac{E}{3(1-2\nu )} \end{aligned},$$

5 $$\begin{aligned} \hat{G}= & {} \frac{E}{2(1+\nu )} \end{aligned},$$

where the Young’s modulus (E) and Poisson’s ratio (${\upnu }$) were input into Abaqus/CAE (DS SIMULIA) as 3250 MPa and 0.3, respectively.

The anisotropic material model uses the Hill’s yield function to define the plastic behaviour of the material. In Cartesian coordinates this is defined as:

6 $$\begin{aligned} f(\sigma ) = \sqrt{F(\sigma _{y} - \sigma _{z})^{2} + G(\sigma _{z} - \sigma _{x})^{2} + H(\sigma _{x} - \sigma _{y})^{2} + 2L\tau _{y z}^{2} + 2M\tau _{z x}^{2} + 2N\tau _{x y}^{2}} \end{aligned},$$

where the constants are given by:

7 $$\begin{aligned} F= & {} \frac{\sigma _{0}^{2}}{2}\left( \frac{1}{\bar{\sigma }^{2}_{22}}+\frac{1}{\bar{\sigma }^{2}_{33}}-\frac{1}{\bar{\sigma }^{2}_{11}}\right) , \\ G= & {} \frac{\sigma _{0}^{2}}{2}\left( \frac{1}{\bar{\sigma }^{2}_{33}}+\frac{1}{\bar{\sigma }^{2}_{11}}-\frac{1}{\bar{\sigma }^{2}_{22}}\right) , \\ H= & {} \frac{\sigma _{0}^{2}}{2}\left( \frac{1}{\bar{\sigma }^{2}_{11}}+\frac{1}{\bar{\sigma }^{2}_{22}}-\frac{1}{\bar{\sigma }^{2}_{33}}\right) \end{aligned},$$

8 $$\begin{aligned} L= & {} \frac{3}{2}\left( \frac{\tau _{0}}{\bar{\tau }_{23}}\right) ^{2}, \\ M= & {} \frac{3}{2}\left( \frac{\tau _{0}}{\bar{\tau }_{13}}\right) ^{2}, \\ N= & {} \frac{3}{2}\left( \frac{\tau _{0}}{\bar{\tau }_{12}}\right) ^{2} \end{aligned},$$

the ratios $\bar{\sigma }_{ij}^{2}/{\sigma }_{0}^{2}$ and $\bar{\tau }_{ij}^{2}/{\tau }_{0}^{2}$ define the ratio of yield stress in the direction *ij* (where *i* and *j* = 1,2,3) to the yield stress in the tabulated stress-strain data used to define the plastic behaviour. The stress-strain data for the circumferential direction was entered into Abaqus/CAE (DS SIMULIA) before the ratio:

9 $$\begin{aligned} \bar{\sigma }_{33}^{2}/{\sigma }_{0}^{2} = 1.6 \end{aligned},$$

was used to scale the plastic yield stresses for the axial direction. The yield stress ratio for the radial and shear directions was assumed to be unity.

**Figure 3:**
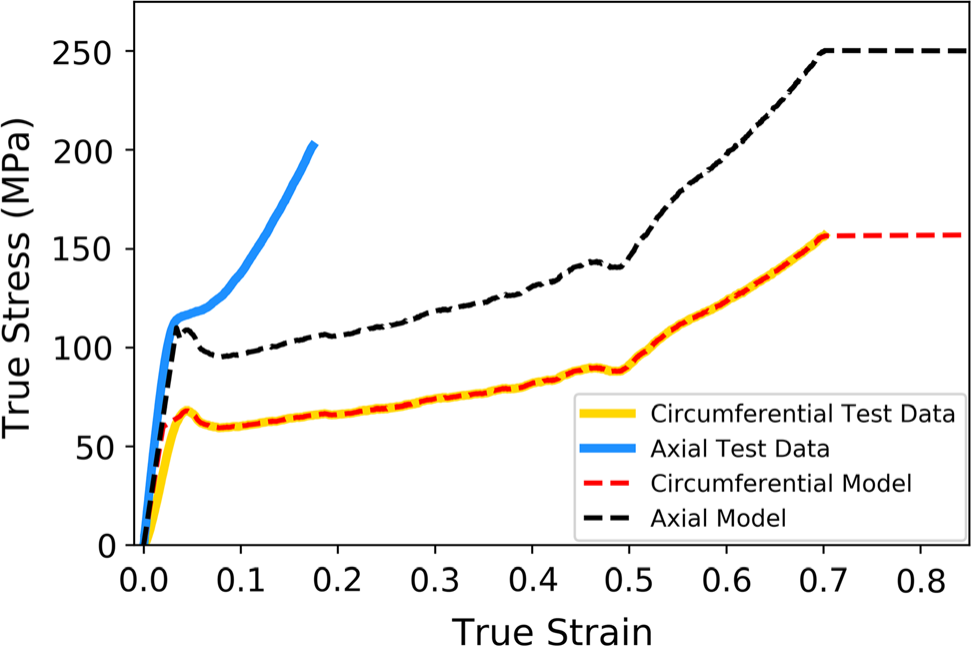
True stress-strain data obtained from uniaxial tensile tests of dogbone shaped specimens cut from die-drawn PLLA tubes compared to the material model utilised in Abaqus (DS SIMULIA). Solid lines represent the data obtained from the material testing whilst the dashed lines show the material properties of the anisotropic model.

Figure 3 shows the stress-strain data obtained from the tensile tests along with the material model implemented in the FEA simulations.

The material properties in the axial direction should be taken into account, as the scaffold crowns undergo bending in expansion and so components of both axial and circumferential deformation are present. However, from previous experience of *in-silico* scaffold expansion, it is clear that the stresses and strains in the axial direction are dominated by those in the circumferential direction. Therefore, the impact of this difference is small due to the limited strains developed in the axial direction.

### Mesh Parameters

#### Scaffold Model

The scaffold was meshed using C3D8R reduced integration elements. These are 8-node 3D stress elements with trilinear shape functions. A mesh refinement study was undertaken where the final diameter and reaction force of the scaffold nodes were found to deviate by less than 4% when refining the mesh from a seed size of 0.04 mm to 0.02 mm, confirming that mesh convergence was achieved. Therefore, a mesh size of 0.04 mm was selected.

#### Folded Balloon Model

A tapered tri-folded balloon model, similar to that used by De Beule et al.,^[12]^ of length 20 mm was used to expand the scaffold. The isotropic elastic material model was used to describe the behaviour of the balloon where E and ${\upnu }$ were taken as 850 MPa and 0.4, respectively. Bilinear 4-node quadrilateral, reduced integration membrane elements were used to mesh the geometry.

#### Crimp Model

A cylindrical surface of length 20 mm was used to crimp and test the specific radial strength of the scaffold. Again, this utilised the isotropic elastic material model where E and ${\upnu }$ were taken as 5000 MPa and 0.3, respectively. The crimping surface was meshed using linear 4-node quadrilateral, reduced integration surface elements.

### Simulation Setup

**Figure 4:**
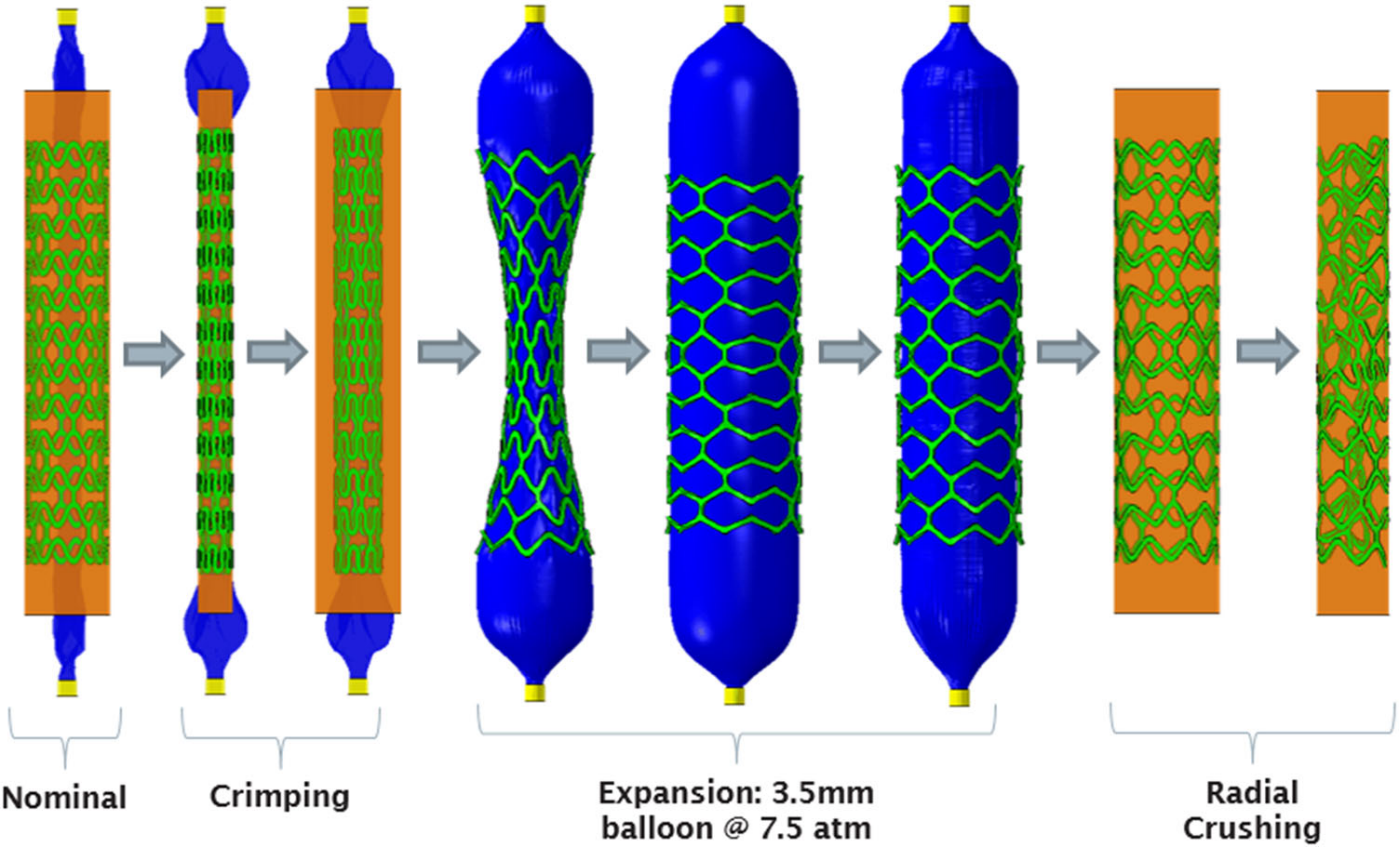
Simulation of the crimping, balloon expansion and radial crushing of the BRS conducted in Abaqus/CAE (DS SIMULIA).

Simulation of the crimping, free expansion and specific radial strength tests was conducted to mimic the mechanical *in-vitro* testing of scaffold platforms conducted by Arterius Ltd (Leeds, UK). This process is depicted in Fig. 4. The simulation comprised the following:

1. Balloon folding. The tapered balloon was wrapped such that it resembled a standard tri-folded balloon.
2. Crimping. The scaffold was crimped from its nominal OD of 2.54 mm to 1.10 mm *via* the displacement driven crimping surface. The surface was then removed to allow the scaffold to recoil.
3. Expansion. The scaffold was expanded by inflating the balloon, pressurised to 0.75 MPa. The balloon was then deflated to allow the scaffold to recoil.
4. Crushing. The scaffold was crushed radially *via* the displacement driven crimping surface to an OD of 2 mm.

The Abaqus/Explicit (DS SIMULIA) solver, as used in many similar studies,^[12,17,29]^ was chosen due to its ability to handle complex contact interactions. Whilst the solution is time dependant, the inertia forces are not dominant and so the simulation can be approximated as a quasi-static process. Therefore, the kinetic energy of the system should remain low throughout the simulation and should not exceed 5% of the internal energy.^[1]^ The step lengths and time increment were taken as 0.06 s and 2e-7 s, respectively, based upon previous experience.

The FEA simulations were submitted to the University of Southampton Iridis 4 high performance computing cluster. Each simulation was run on a 2.6 GHz Sandybridge 16-core node and took approximately 6 hours to complete.

### In-Vitro Mechanical Testing

The *in-vitro* testing referred to herein was conducted by Arterius Ltd (Leeds, UK) on the baseline design (design 5) and previous generations of the ArterioSorb$^{\rm{{TM}}}$. Firstly, the scaffold was crimped incrementally on to a balloon-catheter at $45^{\circ }\,\hbox {C}$ to an OD of 1.1 mm. The scaffold was then expanded in stages using a 3.0 mm diameter tri-folded balloon, inflated to 1.2 MPa, followed by a tri-folded balloon of diameter 3.5 mm, inflated to 0.75 MPa, intended to expand the scaffold to a target OD of 3.8 mm. This was conducted whilst the scaffold was submerged in a water bath, heated to $37^{\circ }\,\hbox {C}$ to simulate the haemodynamic environment. This multi-step process is similar to that used in a clinical scenario where post-dilatation of the scaffold is commonplace. Once the post-dilatation balloon was deflated and removed the scaffold elastically recoiled and was then radially crushed to an OD of approximately 2 mm using a Blockwise TTR2 radial force testing machine. The diameter of the scaffold was measured at each stage of the crimping and expansion process, separately for each scaffold ring using a laser measurement system.

### Performance Metrics

#### Cell Area

Cell area (*CA*) is the space enclosed by a single open cell at the end of the device. The choice of end is arbitrary given the scaffold is axially symmetric.

#### Final Diameter

Final diameter (*FD*) measures the OD of the scaffold once it has elastically recoiled post balloon-expansion. The diameter of an end ring of the scaffold was measured at multiple locations around the circumference to calculate a mean value.

#### Percentage Recoil

Percentage recoil ($R_{\%}$) is defined as the percentage difference between the maximum diameter (*MD*) of an end ring, at the midway point of the expansion step where the balloon reaches the maximum inflation pressure and the final diameter (*FD*) at the end of the expansion step, defined as:

10 $$\begin{aligned} R_{\%} = \left( \frac{ MD - FD }{ MD } \right) \times 100 \end{aligned}.$$

Recoil provides a convenient method of comparing the expected *FD* of the scaffolds given that each design will achieve a different *MD* in the simulations due to their different *SRS*. In clinical practice, the balloon inflation pressure would be varied to ensure to ensure that a deployed scaffold was suitably expanded.

#### Specific Radial Strength

Specific radial strength (*SRS*) is the maximum reaction force of the crimping cylinder divided by the scaffold length. This is defined as:

11 $$\begin{aligned} SRS = \frac{1}{l}\sum _{i=1}^{i=3240} F_{i} \end{aligned},$$

where $F_{i}$ is the reaction force of the $i^{th}$ of the 3240 nodes that constitute the crushing cylinder and *l* is the length of the scaffold.

#### Equivalent Plastic Strain

**Figure 5:**
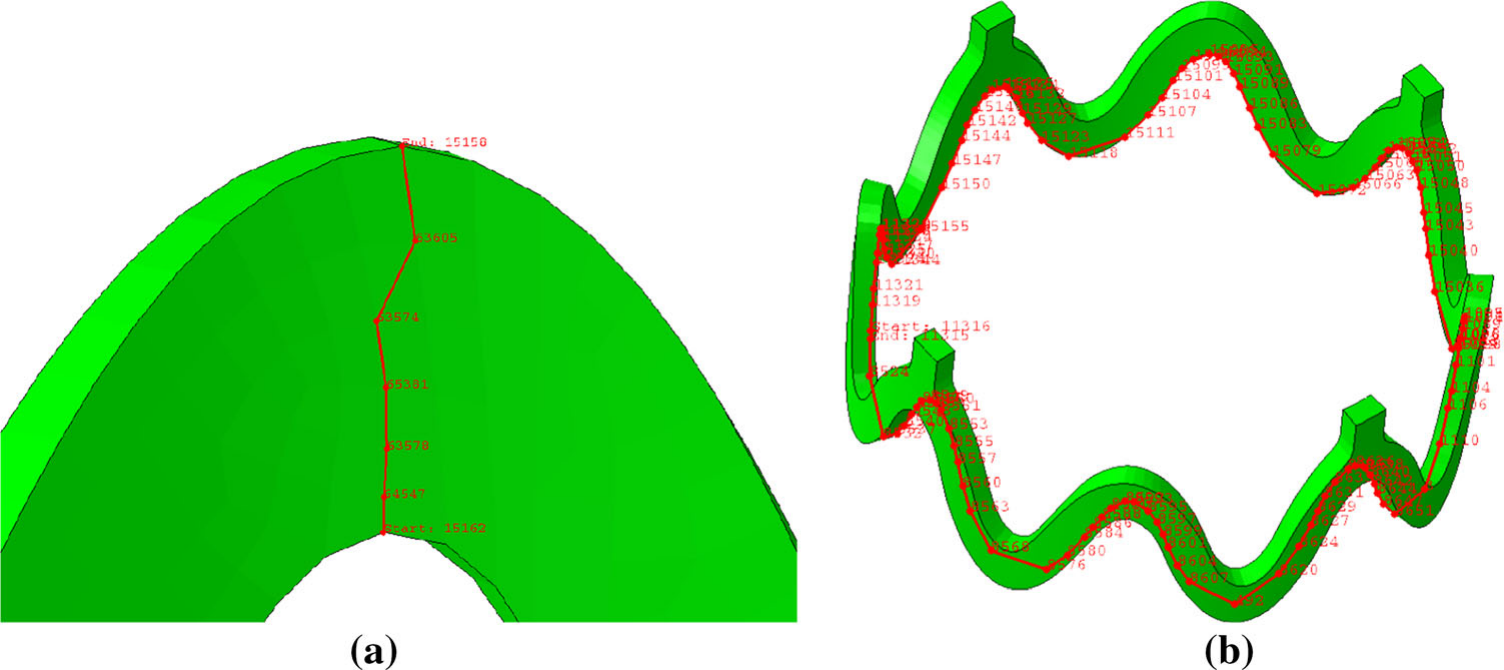
The paths used to inspect PEEQ were located at the scaffold end ring and across the crown width on the inside face of the scaffold where PEEQ is greatest. The crown path, (a) originates at the smaller inside radius of the crown and traverses the width of the strut to the outer radius of the crown. The ring path is shown in (b).

The distribution of *PEEQ* was analysed on paths across each of the crowns’ widths, (Fig. 5a) and along paths around the end ring of the scaffold, (Fig. 5b). The metric, $PQ_{\rm{{max}}}$ is defined as the average of the maximum *PEEQ* at each end ring crown, found using the ring path. An end ring was chosen as this provides the strongest contrast in performance between different designs.

The equivalent plastic strain is defined as:

12 $$\begin{aligned} \bar{\epsilon }^{pl} = \left. {\bar{\epsilon }^{pl}}\right| _{0} + \int _{0}^{t} \dot{\bar{\epsilon }}^{pl} dt \end{aligned},$$

where $\dot{\bar{\epsilon }}^{pl}$ is defined depending upon the material model in use, in this case utilising the Mises definition:

13 $$\begin{aligned} \dot{\bar{\epsilon }}^{pl} = \sqrt{ \frac{2}{3} \dot{\epsilon }^{pl} : \dot{\epsilon }^{pl} } \end{aligned},$$

where $\dot{\epsilon }^{pl}$ is the vector containing the rate of plastic strain in each direction.

**Figure 6:**
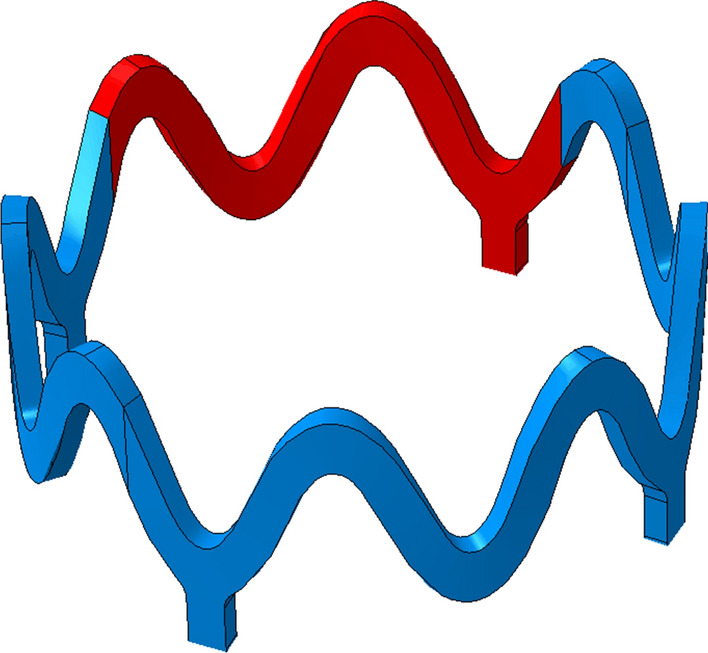
A repeating unit, highlighted red, at the end ring of a scaffold

Due to the circumferential symmetry of the scaffold geometry, the PEEQ data along the scaffold end ring paths was averaged across each of the four repeating units that form an open scaffold ring. Figure 6 shows a repeating unit of the end ring of the scaffold, highlighted red.

To assess the PEEQ across the crown width, a fourth order polynomial regression model was used to fit to the data. Using an average of the eight PEEQ values (one per crown) at each location across the crown width was not possible because of the differences in mesh discretisation between crowns due to the use of a swept mesh. This issue did not affect the ring paths and so an average value at each location along the repeating unit was used to aid visualisation and analysis of the data. The data for the eight crown paths was divided into two groups for model fitting - those with and without a connector attached, as this significantly affected the PEEQ distribution.

## Results

Table 2 shows the performance metrics for each of the five designs simulated. Design 2 has the greatest *SRS* and the smallest *FD*. However, it also presents the largest level of $PQ_{\rm{{max}}}$ at the scaffold end rings and the smallest *CA*. In contrast, design 5 offers a similar level of *SRS* to design 2 yet a greatly reduced level of $PQ_{max}$ and a larger *CA*. This broadly describes the design trade-off that might be expected. Short and wide struts will offer good levels of radial strength and recoil whilst long narrow struts provide improved cell area and plastic strain characteristics. Interestingly, design 2 (a short-wide strut design) appears not to offer a significant benefit over design 5 in terms of *SRS* which is explored below.

**Table 2:** Cell area, final diameter, percentage recoil, specific radial strength and average maximum PEEQ for the 5 designs as well as the *in-vitro* test of the baseline scaffold (design 5).

Design number	Ring length (end ring) (mm) $L_{\rm{{Ring}}}$	Strut width (end ring) (mm) $W_{\rm{{Strut}}}$	Cell area ($mm^{2}$) CA	Final diameter (mm) FD	Recoil $(\%)$ $R_{\%}$	Specific radial strength (N/mm) SRS	Average Maximum PEEQ $PQ_{\rm{{max}}}$
1	0.285	0.179	1.46	3.61	5.25	1.27	0.67
2	0.285	0.268	1.29	3.47	4.14	1.54	1.20
3	0.426	0.179	1.85	3.51	10.23	1.01	0.50
4	0.426	0.268	1.63	3.63	7.87	1.15	1.09
5 (baseline)	0.350	0.220	1.55	**3.57**	**7.27**	**1.50**	0.84
*in-vitro*	0.350	0.220	1.55	**3.71**	**2.62**	**0.89**	n/a

The *in-vitro* test and baseline results are highlighted in bold to aid comparison

**Figure 7:**
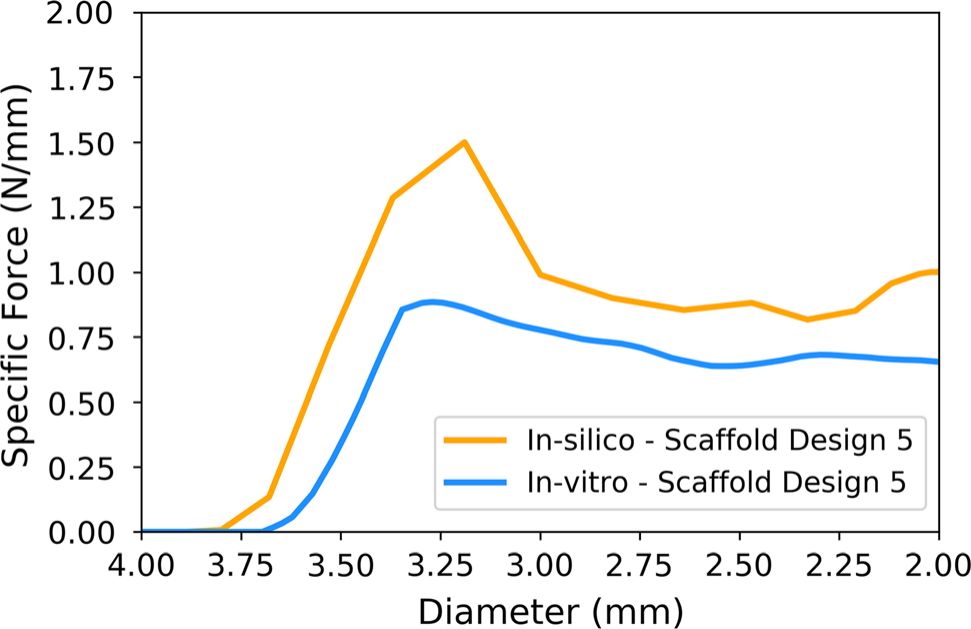
A comparison of the *in-vitro* and *in-silico* force/diameter curves for the radial crushing process.

Figure 7 displays a comparison of the force/diameter curves for the radial crushing step of the *in-vitro* and *in-silico* tests for scaffold design 5. Whilst the stiffness in the two cases is very similar, shown by the initial linear gradient of the curves, the *in-silico* prediction significantly overestimates the maximum force required to crush the scaffold.

**Figure 8:**
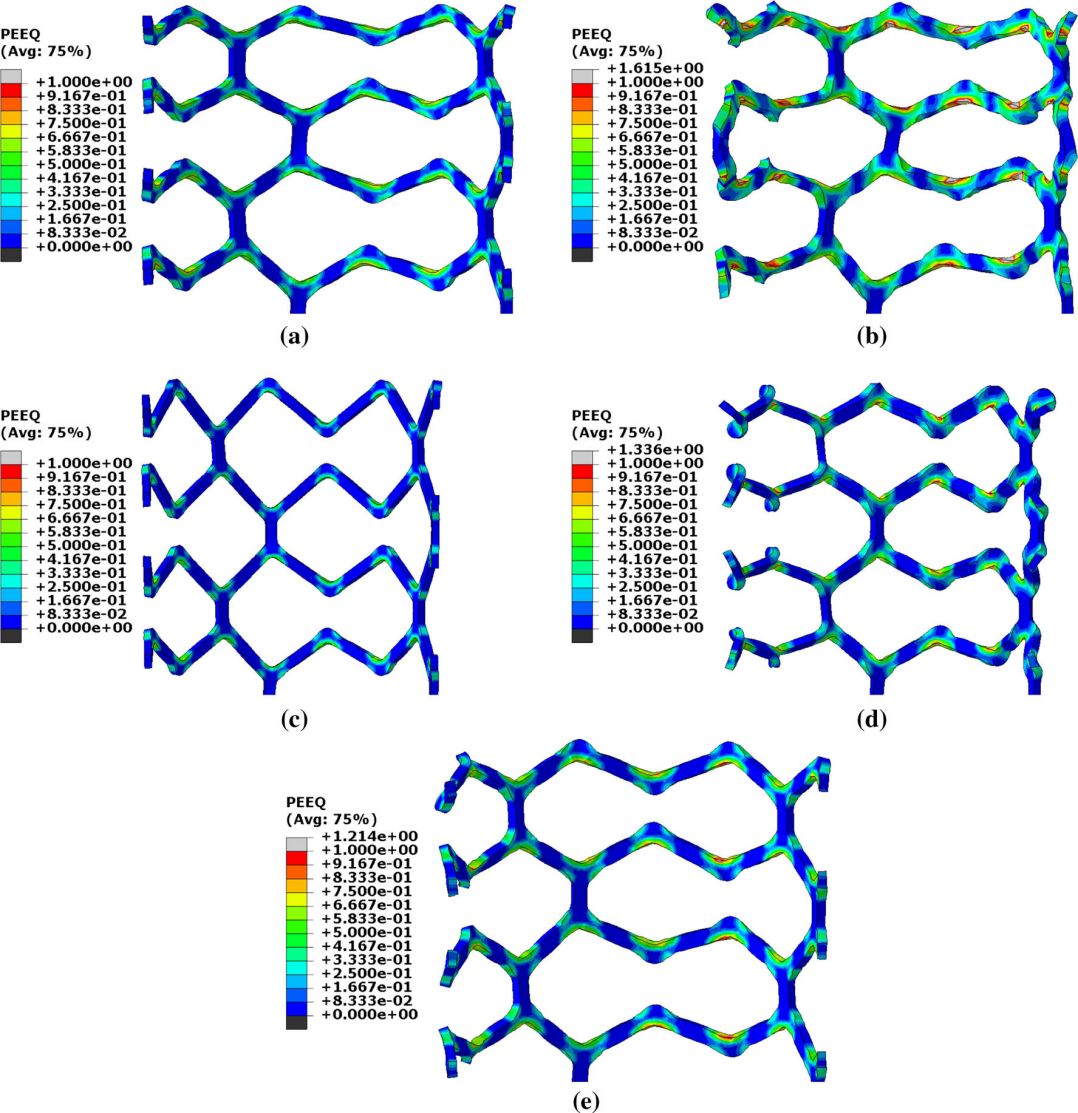
Equivalent plastic strain for each scaffold design at maximum inflation pressure. (a) Design 1, (b) design 2, (c) design 3, (d) design 4 and (e) design 5.

Figure 8 gives the PEEQ distribution in four rings of each of the five scaffolds in expansion when the balloon is at maximum inflation pressure. It is evident that the difference in geometry between each design yields significantly different distributions of PEEQ. The short-wide strut design (design 2, Fig. 8b) shows the greatest level of PEEQ with an average maximum of 1.2 across the eight crowns. Design 2 displays significant splaying of the crowns in the radial direction whilst design 4, (Fig. 8d) shows twisting at the crown apex as the scaffold is expanded. Design 3, (Fig. 8c) displays the lowest level of PEEQ development amongst the five designs where PEEQ at the inside of the crown does not exceed 0.5.

**Figure 9:**
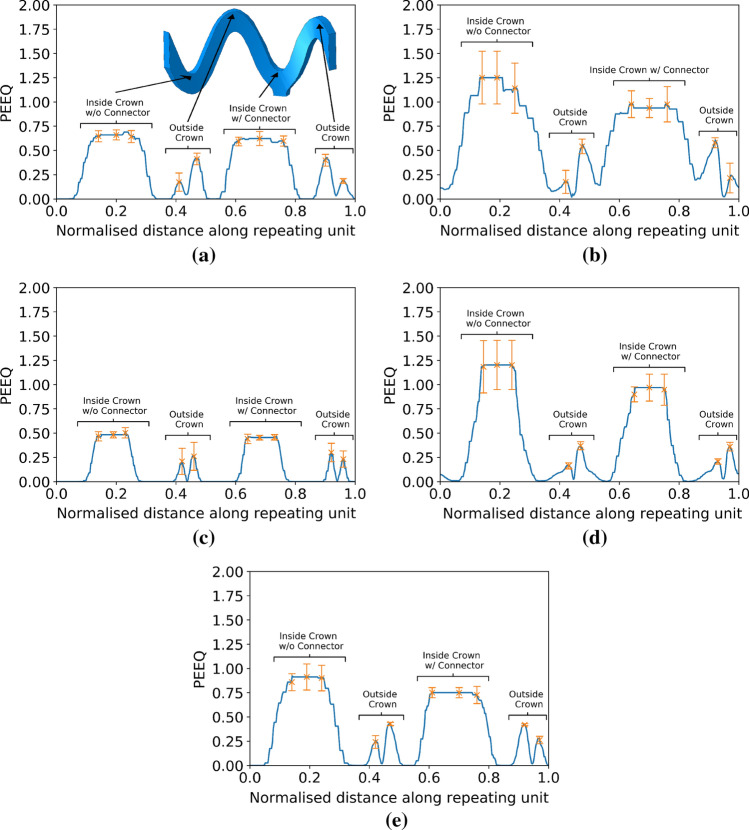
An averaged value of equivalent plastic strain for the four repeating units along paths around an end ring of each scaffold design. (a) Design 1, (b) design 2, (c) design 3, (d) design 4 and (e) design 5 are shown.

Figure 9 details the PEEQ along the path denoted in Fig. 5b, averaged for a single repeating unit, from an end ring of each of the five scaffold designs. The error bars show the maximum deviation of the average value from the data points. Designs 2, 4 and 5 in Fig. 9b, 9d and 9e, respectively, show an alternating pattern of PEEQ at the inside of each crown due to the presence of a connector. This is evident in the two different amplitudes of the largest curves which denote the inside of the crown, where the largest level of PEEQ exists. This effect does not appear to be present in the narrow strut designs, shown in Fig. 9a and 9c, where the curves denoting the inside of the crown are of equal amplitude. In the case of each design, with the exception of design 4, the level of PEEQ on the outside of the crown is symmetrical about the crown attached to a connector. Indeed, the small ‘v’ shaped peaks are of equal amplitude on each side of the large amplitude peak. In the case of design 4, the PEEQ distribution on the outside of the crown is asymmetric about the connector. This is evident *via* the right side of each ‘v’ shaped peak in Fig. 9d displaying a larger value than the left side. It is also evident that the wide strut designs show greater variability in maximum PEEQ compared with the narrow strut designs, evidenced in the larger error bars in Fig. 9b, 9d. Figure 9b also confirms that the PEEQ in design 2 only drops to zero in an extremely small portion of the straight section of the strut.

**Figure 10:**
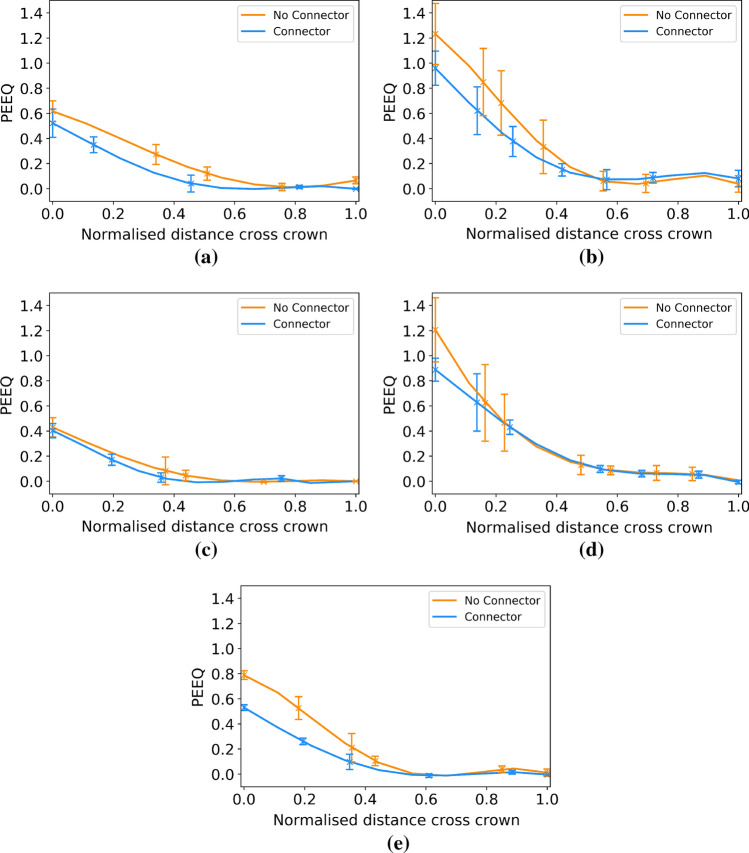
Equivalent plastic strain across the crown width of an end ring of each scaffold design for crowns with and without a connector. (a) Design 1, (b) design 2, (c) design 3, (d) design 4, and (e) design 5.

Figure 10 displays the values of PEEQ across the two groups of crowns on the end ring of each scaffold design using the path in Fig. 5a. As expected from Figs. 8 and 9, designs 2 and 4 display significantly greater levels of PEEQ at the inside of the crown and show a greater level of penetration of PEEQ across the crown. As per Fig. 9, the presence of a connector reduces the level of PEEQ developed at the inside of the crown. This is particularly evident in design 5, (Fig. 10e) where there is a large difference in maximum PEEQ between the two cases. This is also present in designs 2 and 4 but less noticeable, in part due to the large variability of PEEQ.

## Discussion

Firstly, a comparison of the *in-silico* and *in-vitro* tests for scaffold design 5 shows that the simulations poorly predict the scaffold’s *SRS* and $R_{\%}$, predominantly attributed to the constitutive material model. However, the *FD* of the scaffold predicted by the model is 3.57 mm, compared to 3.71 mm according to the *in-vitro* test which gives a percentage error of less than 4%. Whilst an elasto-plastic model utilising the Hill’s yield function has been employed in previous computational studies of polymeric BRS,^[7,31]^ Hoddy *et al*.^[18]^ have demonstrated the challenge of accurately predicting both the elastic recoil and radial strength of a scaffold based upon the thin-strut ArterioSorb$^{\rm{{TM}}}$ using this model framework. However, their novel user-defined material model predicted radial strength within 1.1% of the analogous *in-vitro* test for only a small reduction in accuracy of the post-expansion diameter prediction. To date, more advanced material models that capture the viscous response of PLLA have been used to explore polymeric BRS in the context of FEA.^[5,8,9]^ However, viscoelastic-plastic models that utilise a parallel network rheology require a greater effort in terms of calibration to the uniaxial tensile data and those available in Abaqus/Explicit do not allow for any description of anisotropy which appears critical in the PLLA used to construct the ArterioSorb$^{\rm{{TM}}}$. Eswaran *et al*.^[13]^ very accurately predicted the force/displacement profile of a single ring early generation BRS when subjected to radial expansion and crushing by employing a user-defined anisotropic viscoelastic-plastic material model. Therefore, whilst the elasto-plastic material model employing the Hill’s yield function represents a compromise, it was considered beyond the scope of this research to develop an alternative material model that can more accurately describe the material anisotropy and capture its viscous time-dependant behaviour. However, it is evident from the literature that improvements in material modelling will result in more accurate predictions of scaffold mechanical behaviour.

**Figure 11:**
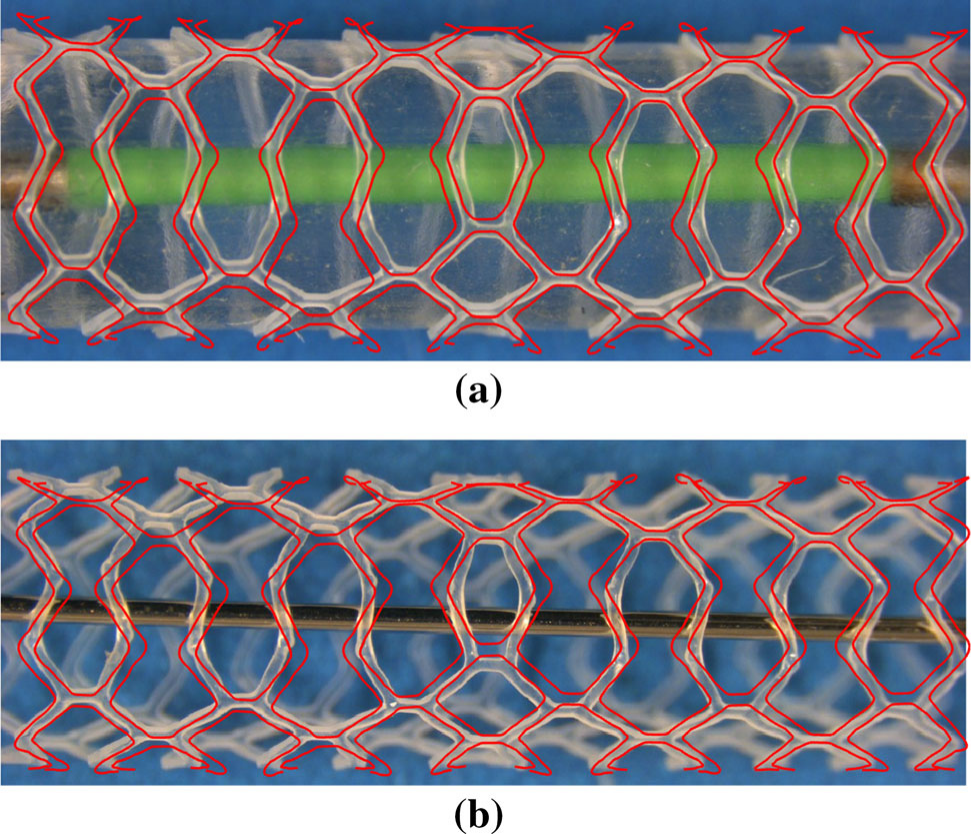
Comparisons of the scaffold shapes for (a), the diameter at maximum balloon inflation pressure and (b), the final recoiled diameter.

Figure 11 shows a comparison of the *in-silico* and *in-vitro* expansion of scaffold design 5 at the maximum and final diameters. The *in-silico* scaffold is shown as a red overlay upon an image of the *in-vitro* test. Observing Fig. 11b, it can be seen that the *in-vitro* scaffold elastically recoils less than the *in-silico* scaffold, as per Table 2, as the open cell crowns remain more greatly straightened. Whilst prediction of the final diameter yields less than a 4% error, the maximum diameter is predicted with even greater accuracy as 3.85 mm compared to 3.81 mm as reported by the *in-vitro* test, an error of 1%. Differences are noticeable in the scaffold shapes, particularly in Fig. 11b at the central closed ring. In addition to the limitation of the material model, this discrepancy could also be a result of the interaction between the scaffold and balloon. The *in-vitro* test results in the crimped scaffold remaining firmly in contact with the balloon after the crimp is removed whilst the *in-silico* test leaves a small gap between the crimped scaffold and balloon. Moreover, the friction between the scaffold and balloon is set using a friction coefficient of 0.1 yet this value is difficult to validate and may be a source of error. The scaffold also appears to bend slightly in the *in-vitro* test which results in the left hand rings of the *in-silico* scaffold misaligning with the *in-vitro* scaffold in Fig. 11b. However, prediction of the scaffold shape generally appears most accurate at the end rings, the location at which the values of *PEEQ* are extracted. This gives improved confidence regarding the prediction of the local strains at this critical location.

It is evident in Fig. 7 that the scaffolds display a similar level of initial stiffness in the radial crushing process but the *in-silico* prediction significantly overshoots the maximum force required to crush the scaffold from its recoiled diameter. In Fig. 7 the force/diameter curves are displaced from each other in the x direction due to the scaffolds reaching different final diameters leading to the crimp making contact with the scaffold at different points in the two tests. After the maximum force is applied the scaffold is considered to have failed. Therefore, predicting the force/diameter relationship after this point it less critical than predicting the maximum force and stiffness.

Observation of the five scaffold designs makes clear that variations in ring length and strut width, of approximately +/- 20% from the baseline design, along the scaffold length yield a change in mechanical performance, as per Table 2. This results in *SRS* ranging from 1.01 N/mm to 1.54 N/mm and $R_{\%}$ varying from 4.14% to 10.23%. There is also a significant variation in $PQ_{\rm{{max}}}$, from 0.67 to 1.20. Referring to Table 2, short-wide strut designs afford improved levels of *SRS* and $R_{\%}$ due to the increased levels of PEEQ developed at the crown apex whilst long-narrow strut designs naturally accommodate superior side branch access with a reduced risk of strut fracture due to the lower levels of plastic deformation.

**Figure 12:**
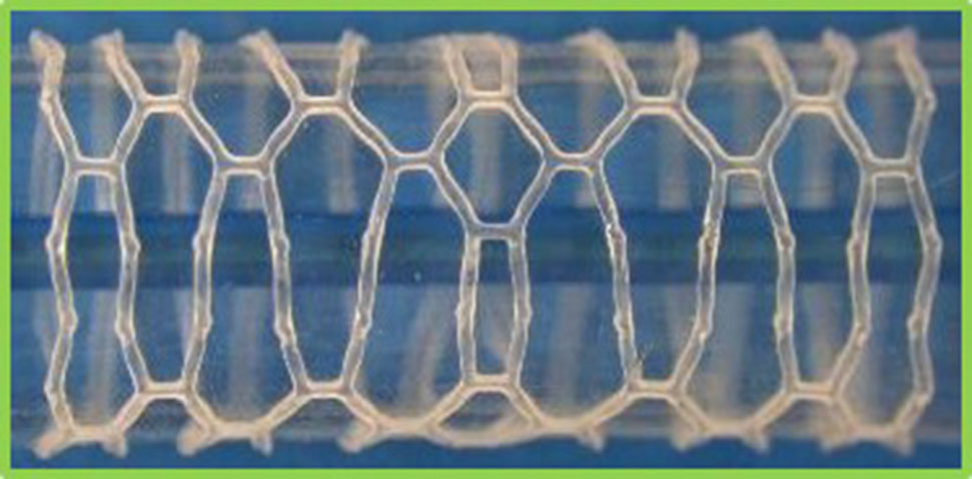
Design 5 over-expanded to an outer diameter of 4.39 mm without evidence of strut fracture occurring.

The use of defined paths upon which to observe the PEEQ, significantly aids the quantification and analysis of the subtle differences in PEEQ distribution between each scaffold. Designs 1 and 5 show similar distributions of PEEQ, according to Fig. 8. This is unsurprising given they both have the same ratio of ring length to strut width. Design 1 has a 15% lower *SRS* than design 5, due to the lower level of PEEQ developed at the crown apex, evidenced in Fig. 10 where the $PQ_{\rm{{max}}}$ is approximately 0.6 for design 1, compared with 0.8 for design 5. Interestingly, in the case of design 5, $PQ_{\rm{{max}}}$ is greater than the ultimate tensile strain (UTS) of PLLA, which is 0.7 in the circumferential direction, as per Fig. 3. This indicates the ability of the scaffold to tolerate large levels of plastic strain in expansion. Figure 12 strengthens this argument as it shows design 5 significantly over-expanded without displaying evidence of strut fracture. Many of the crowns appear to have been completely straightened yet do not display any whitening of the plastic known as ’crazing’, first observed in BRS by Radu *et al*.^[33]^ Indeed, this validates design 5, and, by extension, design 1, as viable designs that tolerate significant plastic strain, even when over-expanded, as is commonplace in clinical practice.^[15]^ Figure 10(a) and 10(e) show two clear patterns of PEEQ penetration across the crown width. It is evident that the presence of a connector attached to a crown restricts the ability of the crown to open in expansion, limiting the development of plastic strain. This will result in crowns in any given scaffold ring displaying alternating stress levels which could worsen uneven resorption of the scaffold where resorption occurs fastest at the highly stressed crowns.^[25,35]^ This in turn could exacerbate the long term blood clotting risk if scaffold struts protrude into the blood flow, providing sites for thrombus formation. Therefore, the use of a closed cell scaffold design, whilst reducing the cell area afforded by the scaffold, may improve the likelihood of even resorption of BRS.

In contrast to designs 1 and 5, design 3 displays a very low level of plastic strain developed in the scaffold struts. According to Figs. 8 and 9, the value of $PQ_{\rm{{max}}}$ does not exceed 0.5. Figure 10(c) shows that the PEEQ does not penetrate far into the straight sections of the strut, leading to the poor $R_{\%}$ and *SRS* performance in design 3. However, this design does have a large *CA* to facilitate improved side branch access. The presence of a connector does not appear to influence the PEEQ distribution in the narrow strut design. Referring to Fig. 10c there is no difference in the $PQ_{\rm{{max}}}$ between the two groups, most likely due to the scaffold being under expanded for its ring length.

**Figure 13:**
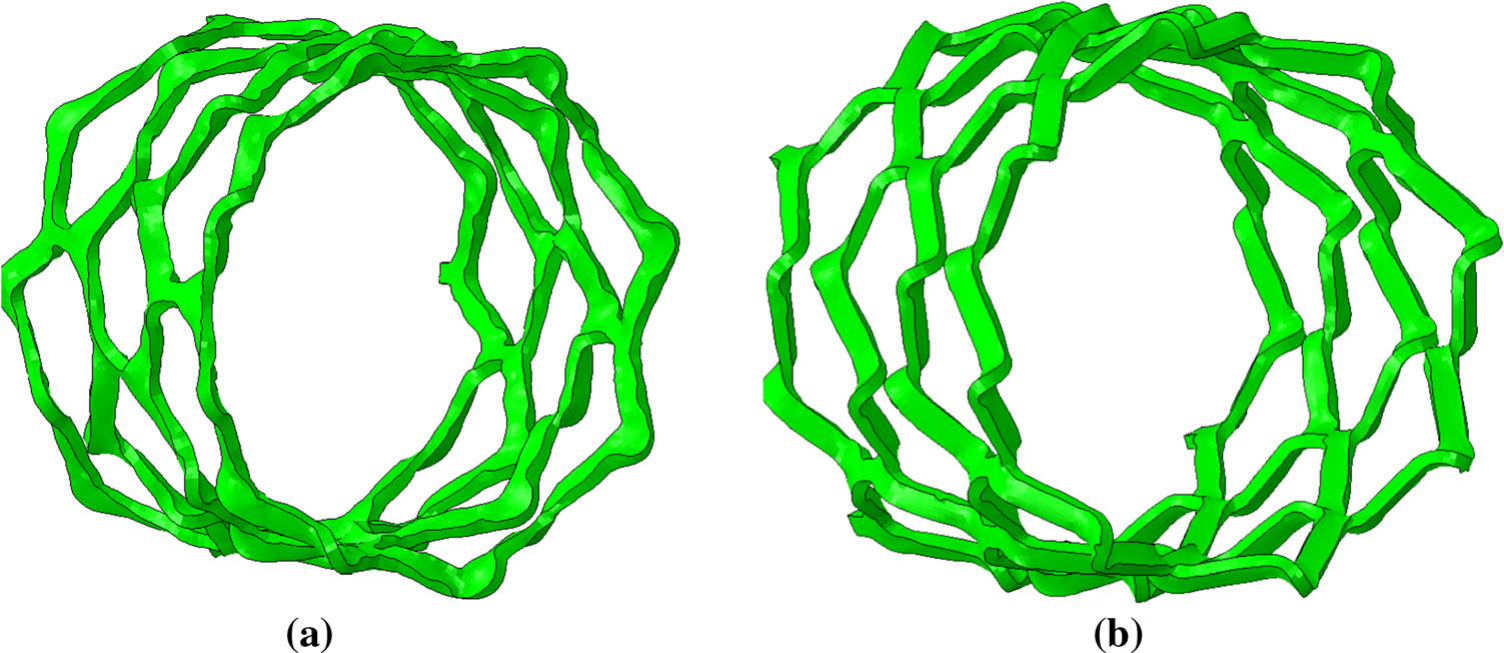
Wide-strut scaffold designs that exhibit twisting when expanded. (a) Design 2, shows some splaying of the crowns in the end ring whilst (b) design 4 displays significant twisting in each of the four rings shown.

**Figure 14:**
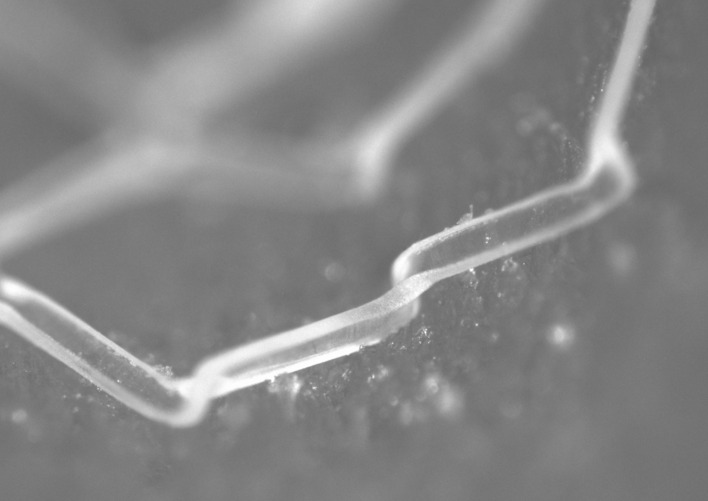
*In-vitro* evidence of strut twisting about the crown apex in the deployment of a long ring-length BRS into a mock silicon vessel. The initial scaffold expansion was conducted by Arterius Ltd whilst the image was obtained using an optical microscope at the University of Southampton’s material laboratory.

Referring to the wide strut designs (designs 2 and 4), significantly more plastic strain is developed in the scaffold struts compared to the narrow strut designs. In addition to the reduced radius of curvature at the inside of the crown apex, this is due to the twisting and splaying of the crowns that occurs in expansion, highlighted in Fig. 13 which shows designs 2 and 4 at maximum balloon inflation pressure. This behaviour is likely to occur due to the high ratio of strut width to strut thickness. As the scaffold is expanded, the conventional cantilever movement in the crown is exchanged for twisting in the radial direction as this provides less resistance to open the scaffold ring. The twisting behaviour has also been observed in *vitro* when an alternative long ring-length scaffold design was deployed into a silicon coronary artery model, shown in Fig. 14. Whilst any twisting or splaying of the scaffold struts may be expected to be constrained by the vessel wall in *vivo*, this does not appear to be the case in *vitro*. The tendency of the struts to twist will heighten the stress exerted on the arterial lining, increasing the risk of neointimal hyperplasia,^[14,24]^ as well as increasing the risk of thrombus formation due to malapposed scaffold struts. Of course, reducing stress exerted on the vessel wall is highly desirable. Indeed, this is highlighted by a number of studies that assess the stress exerted by coronary stents/scaffolds on the arterial layers.^[28,30,32]^ This mechanism also explains the relatively poor *SRS* performance of design 4 as the weaker resistance of the struts in the radial direction is exposed to the crushing force, rather than utilising a component of the stiffer material properties in the axial direction.

Observing Fig. 9(b) and 9(d) it can be seen that the $PQ_{\rm{{max}}}$ in designs 2 and 4 at the inside of the crown apex varies by approximately +/- 20% between crowns, highlighted by the error bars in both Figs. 9 and 10. It is likely the splaying and twisting of the crowns results in the large variation in maximum PEEQ, in part due to the small lateral movement of the location of $PQ_{\rm{{max}}}$. Twisting at the crown apex results in the location of maximum PEEQ moving away from the geometrical centre of the crown, leading to the $PQ_{\rm{{max}}}$ values in each repeating unit not aligning. Moveover, the very large rate of change of PEEQ at the inside of the crown apex also leads to differences between each repeating unit.

**Figure 15:**
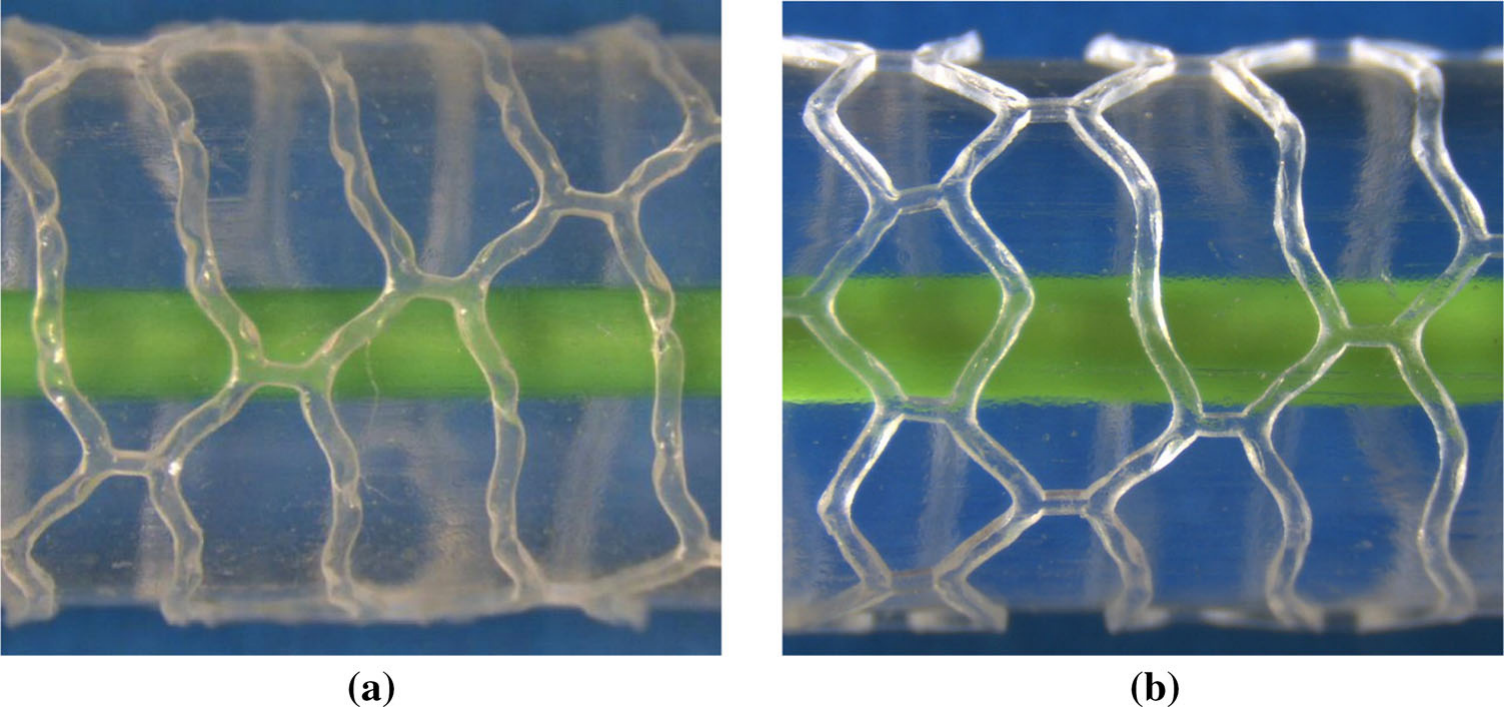
A comparison of the penetration of equivalent plastic strain across the width of crowns. In (a) this is visible due to the whitening effect of the plastic. This effect has been termed hinging and leaves the scaffold vulnerable to fatigue failure. In comparison, (b) shows no evidence of hinging.

Significantly, only a very small portion of the scaffold struts in design 2 appear to have developed no plastic strain. This is confirmed by Figs. 8b and 9b where the PEEQ has penetrated far into the straight sections of the struts. Although design 2 provides good *SRS* performance (albeit only marginally higher than design 5) it does present a significant risk of strut fracture, due to the high level of $PQ_{\rm{{max}}}$. Referring to design 4 in Fig. 9d, it is evident that the PEEQ is contained more closely to the vicinity of the crown apex, compared to design 2. This explains the relatively low level of *SRS* in design 4. However, design 4 still presents a significant risk of strut fracture due to the large value of $PQ_{\rm{{max}}}$ developed at the inside of the crown. Figure 10b shows that a significant level of PEEQ develops to around 50% of the crown width and some PEEQ even penetrates the entire crown width. This can be extremely detrimental to the scaffold as it can induce the ’hinging’ phenomenon, whereby plastic strain visibly penetrates across the width of the crown, evidenced by the whitening of the plastic from the inner to outer crown radius. Figure 15 shows two different previous generations of the ArterioSorb$^{\rm{{TM}}}$ BRS, which utilise a helical pattern of connectors, expanded *via* balloon inflation, one of which, depicted in Fig. 15a, shows the onset of ’hinging’ even when expanded to a modest target diameter. This contrasts the scaffold in Fig. 15b which shows no evidence of hinging when expanded to a similar target diameter. Whilst hinging has not been observed to initiate brittle fracture in scaffolds when expanded, it does leave them extremely vulnerable to fatigue failure and will, as previously mentioned, have implications on the rate at which the scaffold resorbs in the crown region. This in turn could lead to protrusion of the scaffold struts into the lumen presenting an increased risk of thrombus formation.

## Conclusions

Variable ring length and strut width scaffolds were explored *via* *in-silico* testing to facilitate improved over expansion tolerance and side branch access of BRS. Comparison with *in-vitro* data was also conducted to validate the simulations and deepen understanding of the adverse effects that manifest in BRS expansion. Particular attention was given to the development of equivalent plastic strain, PEEQ, in the scaffold struts. This was also observed along defined paths both across the crown widths and along the edges of the scaffold end rings which highlighted a number of subtle differences in PEEQ distribution between each design.

The following conclusions could be drawn:

1. The maximum and final diameters of the scaffold post balloon expansion were accurately predicted by the *in-silico* test with a percentage error of less than 4%. The specific radial strength and percentage recoil of the scaffold was poorly predicted by the *in-silico* testing due to the limitations of the elasto-plastic material model and the difficulty in accurately capturing the interaction between the balloon and scaffold.
2. Wide-strut scaffolds offer a significantly increased risk of brittle strut fracture due to the development of high levels of equivalent plastic strain at the inner radius of the crown. The onset of hinging may also occur in wide-strut designs if equivalent plastic strain penetrates the width of the crown. Allied to this consideration is the radial twisting about the crown apex that occurs; a result of the high strut width to strut thickness ratio. This heightens the risk of damage to the vessel wall, reduces the scaffold’s radial strength and was also found to result in an asymmetric distribution of *PEEQ* either side of the connector.
3. *In-vitro* experiments suggest that the ArterioSorb$^{\rm{{TM}}}$ BRS can tolerate significant over expansions without evidence of brittle fracture or the hinging phenomenon.
4. The presence of a connector attached to a crown will reduce the equivalent plastic strain developed at that crown and alter its distribution around the scaffold ring. This is particularly the case in over-expansion scenarios and for wide strut designs. This will impact the likelihood of brittle fracture, the manifestation of the hinging phenomenon as well as have implications for the even resorption of the scaffold *in-vivo*.
